# Plk1 phosphorylation of CAP-H2 triggers chromosome condensation by condensin II at the early phase of mitosis

**DOI:** 10.1038/s41598-017-05986-7

**Published:** 2017-07-17

**Authors:** Yuya Kagami, Masaya Ono, Kiyotsugu Yoshida

**Affiliations:** 1 Department of Biochemistry, Jikei University School of Medicine, 3-25-8 Nishi-shinbashi, Minato-ku, Tokyo 105-8461 Japan; 2 Division of Chemotherapy and Clinical Research, National Cancer Center Research Institute, 5-1-1 Tsukiji, Chuo-ku, Tokyo 104-0045 Japan

## Abstract

Condensin complexes play crucial roles in chromosome condensation that is a fundamental process to establish the “rod-like” shape of chromosome structure in mitosis. Failure of the chromosome assembly causes chromosome segregation errors and subsequent genomic instability. However, a molecular mechanism that controls condensin function for the chromosomal organization has not been fully understood. Here, we show that the abundance of CAP-H2, one of the condensin II subunits, is fluctuated during the cell cycle in accordance with Plk1 kinase activity. Inhibition of Plk1 leads to Cdc20-mediated degradation of CAP-H2 in mitosis. Plk1 phosphorylation of CAP-H2 at Ser288 is required for the accumulation of CAP-H2 and accurate chromosomal condensation during prophase. These findings suggest that Plk1 phosphorylation regulates condensin II function by modulating CAP-H2 expression levels to facilitate proper mitotic chromosome organization.

## Introduction

Chromosomal morphology is drastically changed during the cell division cycle. The “rod-like” shape of mitotic chromosome structure enables discrimination of the individual chromosome. Disruption of mitotic chromosome architecture impediments chromosome alignment and segregation. Therefore, proper mitotic chromosome structure is required for natural cell proliferation to maintain chromosomal stability. To establish mitotic chromosome organization, chromosome condensation is an essential process and is induced at the initial phase of mitosis. Condensin, a highly conserved protein complex from yeast to vertebrate, plays pivotal roles for chromosomal condensation^1^. In vertebrates, two types of condensin, condensin I and condensin II, have been discovered. A condensin complex forms pentamer and the SMC2-SMC4 heterodimer is shared by condensin I and II as a core component. By contrast, the three non-SMC subunits are composed of different proteins: CAP-H, CAP-G, and CAP-D2 for condensin I; and CAP-H2, CAP-G2, and CAP-D3 for condensin II. Although both condensin I and II have the structural similarities, they are controlled by distinct mechanism. Previous studies have reported that condensin subunits are highly phosphorylated during mitosis and several mitotic kinases regulate condensin functions^2^. In this context, Cdk1 and Plk1 phosphorylate condensins and induce chromosome condensation^3–6^. Aurora B phosphorylation of CAP-H is required for condensin I loading onto mitotic chromosome^7^. In contrast, Mps1-mediated phosphorylation of CAP-H2 regulates chromosomal localization of condensin II^8^. These studies have demonstrated that condensin’s activity and localization are tightly controlled by the phosphorylation of their subunits during mitosis; however, a mechanism that regulates the expression levels of condensin is largely unclear. Although a previous study has reported that the abundance of condensin I subunits, including SMC2 and SMC4, are almost unchanged through the cell cycle progression^9^, whether expression levels of condensin II subunits are fluctuated during the cell cycle remains to be elucidated. Of note, in interphase of Drosophila cells, CAP-H2 protein levels are regulated by Skp-Cullin-F-box (SCF) ubiquitin E3 ligase-mediated degradation, but the SCF target domain of CAP-H2 is found only in Drosophila^10^. This finding implies the possibility that the ubiquitination is involved in regulating condensin II function in other organisms; however, there is no evidence that the subunits of condensin II are regulated by ubiquitin-proteolysis mechanism besides Drosophila.

Ubiquitin-proteasome machinery plays crucial roles in driving the cell cycle. Anaphase-promoting complex (APC) E3 ubiquitin ligase is one of the essential E3 ubiquitin ligase for regulating the cell cycle progression. The APC forms two distinct E3 ligase complexes, APC/Cdc20 and APC/Cdh1, by binding with the substrates-recognizing proteins, Cdc20 and Cdh1, respectively^11^. APC/Cdc20 has indispensable roles in mitotic chromosome segregation during the metaphase to anaphase transition by ubiquitin-mediated destruction of cyclin B and securin^12^. Therefore, depleting Cdc20 leads to mitotic arrest and subsequently induces mitotic cell death^13, 14^. Accumulating evidences have demonstrated that Cdc20 is required for regulating mitotic chromosome behaviors, including chromosome segregation; however, its function in mitotic chromosome organization, such as chromosome condensation, is largely unclear.

Here, we found that the abundance of CAP-H2 is increased in mitosis by a Plk1 kinase activity-dependent manner and that inhibition of Plk1 induces a degradation of CAP-H2 through Cdc20-mediated ubiquitin-proteasome machinery. We concluded that the expression levels of CAP-H2 are regulated by Plk1 and Cdc20 for proper chromosomal organization during mitosis.

## Results

### CAP-H2 protein levels are regulated by Plk1

**Figure 1:**
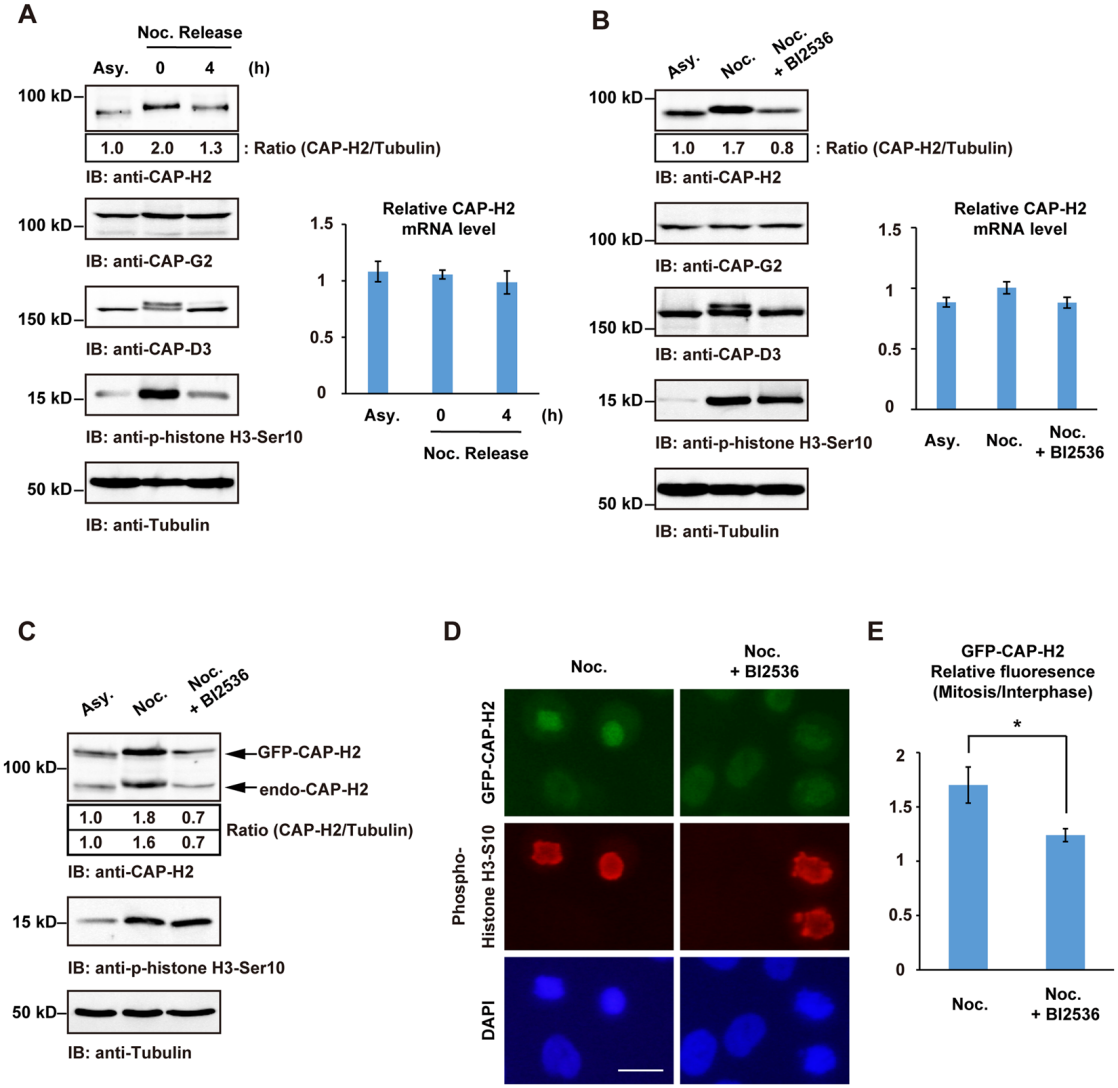
The abundance of CAP-H2 is regulated by Plk1 during mitosis. (**A**) HeLa cells were left untreated (Asy.) or treated with nocodazole (Noc.). Mitotic arrested cells were then released for the indicated times. Cell lysates were immunoblotted with the indicated antibodies. A ratio of CAP-H2/Tubulin was determined by the densitometric analysis using Fusion-CAPT-Software (Vilber Lourmat). The ratio of CAP-H2/Tubulin in asynchronous cells was defined as 1.0. Total RNA was analyzed by real-time RT-PCR with CAP-H2-specific primer. The value is normalized to GAPDH. Data represent mean ± SD from three independent experiments. (**B**) HeLa cells were arrested at mitosis by nocodazole with or without BI2536. Cell lysates were subjected to immunoblot analysis with the indicated antibodies. A ratio of CAP-H2/Tubulin was determined by the densitometric analysis using Fusion-CAPT-Software. The ratio of CAP-H2/Tubulin in asynchronous cells was defined as 1.0. Total RNA was analyzed by real-time RT-PCR with CAP-H2-specific primer. The value is normalized to GAPDH. Data represent mean ± SD from three independent experiments. (**C**) GFP-CAP-H2 stable cell lines were arrested by nocodazole with or without BI2536. Cell lysates were analyzed by immunoblotting with the indicated antibodies. A ratio of GFP-CAP-H2 or CAP-H2/Tubulin was determined by the densitometric analysis using Fusion-CAPT-Software. The ratio of GFP-CAP-H2 or CAP-H2/Tubulin in asynchronous cells was defined as 1.0. (**D**) GFP-CAP-H2 stable cell lines were arrested by nocodazole with or without BI2536, and then fixed. Fixed cells were stained with anti-phospho-histone H3-Ser10. DNA was stained by DAPI. Scale bar, 20 μm. (**E**) The mean fluorescence of mitotic GFP-CAP-H2 was normalized by fluorescence of interphase GFP-CAP-H2. n > 30 mitotic cells per indicated condition were analyzed. Data represent mean ± SD from three independent experiments. Statistical analysis was performed with the Student’s t test (*P < 0.05).

To analyze expression levels of condensin II subunits during the cell cycle, HeLa cells were treated with nocodazole followed by the release from mitosis. Immunoblot analysis revealed that CAP-H2 is increased in mitotic cells (Fig. 1A and Supplemental Fig. S1A). Notably, the abundance of other condensin II subunits, CAP-G2 and CAP-D3, were not fluctuated during the cell cycle (Fig. 1A). Analysis of real-time RT-PCR showed that CAP-H2 mRNA levels are not significantly changed (Fig. 1A and Supplemental Fig. S1B), indicating that the expression of CAP-H2 is fluctuated by post-transcriptional modification(s). To confirm that CAP-H2 is increased during normal mitotic progression, HeLa cells were synchronized at G1/S phase by thymidine and then released into cell cycle. Mitotic cells were collected and cell lysates were analyzed by immunoblot analysis. The results indicated that CAP-H2 is increased in mitotic cells (Supplemental Fig. S1C). A previous study has demonstrated that the condensin II subunits are highly phosphorylated by Plk1 kinase^3^. In this context, we next examined whether Plk1 regulates the abundance of CAP-H2 in mitosis. Inactivating endogenous Plk1 by the pharmacological Plk1 inhibitor, BI2536, led to a reduction of CAP-H2 at protein levels but not at mRNA levels during mitosis (Fig. 1B). Similar results were obtained in RPE1 cells (Supplemental Fig. S1D). Although the expression of CAP-G2 and CAP-D3 remained unchanged (Fig. 1B), as consistent with a previous report^3^, the phosphorylated form of CAP-D3 was detected as a slow migration and the band shift was diminished by BI2536 treatment (Fig. 1B). Depletion of Plk1 by siRNA also suppressed the mitotic CAP-H2 expression (Supplemental Fig. S1E). These results suggest that the abundance of CAP-H2 is controlled by Plk1 kinase activity during mitosis. To investigate that Plk1 regulates the expression of CAP-H2 at post-transcriptional levels, we generated a cell line that stably expresses GFP-tagged CAP-H2 (GFP-CAP-H2). In this cell line, the transcription of GFP-CAP-H2 mRNA was regulated by the promoter that was derived from transfected plasmids. Thus, endogenous and exogenous CAP-H2 mRNA expressions were controlled by distinct mechanisms. Immunoblot analysis showed that both endogenous and exogenous expressions of CAP-H2 are elevated during mitosis (Fig. 1C). Furthermore, Plk1 inhibition prevented the accumulation of both endogenous CAP-H2 and GFP-CAP-H2 in mitosis (Fig. 1C). Immunofluorescence analysis also showed that BI2536 treatment reduces GFP-CAP-H2 in mitotic cells (Fig. 1D and E). These results demonstrate that the abundance of mitotic CAP-H2 is regulated by Plk1 kinase activity at post-transcriptional levels.

**Figure 2:**
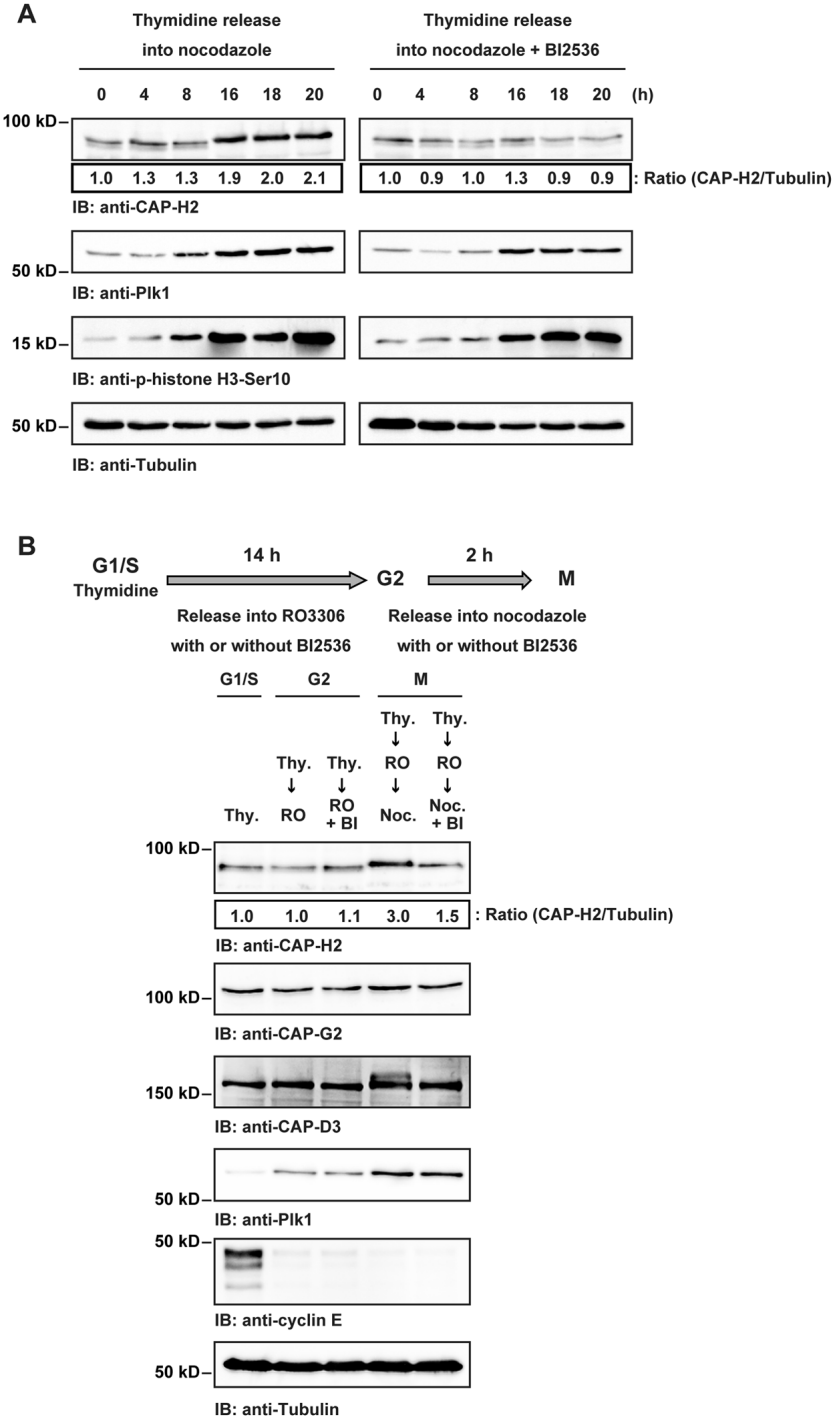
Plk1 kinase activity is required for regulating CAP-H2 protein levels. (**A**) HeLa cells were synchronized by double thymidine block. The cells were released into medium containing nocodazole with or without BI2536 for the indicated times. Cell lysates were immunoblotted with the indicated antibodies. A ratio of CAP-H2/Tubulin was determined by the densitometric analysis using Fusion-CAPT-Software. The ratio of CAP-H2/Tubulin in 0 h was defined as 1.0. (**B**) HeLa cells were arrested at G1/S phase by double thymidine block. To synchronize at G2 phase, the cells were released into media containing RO3306 with or without BI2536 for 14 h. G2 phase arrested cells were released into nocodazole with or without BI2536 for 2 h. Cell lysates were analyzed by immunoblotting with indicated antibodies. A ratio of CAP-H2/Tubulin was determined by the densitometric analysis using Fusion-CAPT-Software. The ratio of CAP-H2/Tubulin in thymidine treated cells (G1/S) was defined as 1.0.

To further investigate the fluctuation of CAP-H2, HeLa cells were arrested at G1/S by a double thymidine block. The cells were released into media containing nocodazole with or without BI2536 and cell lysates were subjected to immunoblot analysis. The results indicated that the abundance of CAP-H2 correlates with Plk1 and is increased during mitosis (Fig. 2A). To confirm these results, HeLa cells were synchronized at G1/S, G2 or mitosis using cell synchronization methods in the presence or absence of Plk1 inhibitor (Fig. 2B). Immunoblot analysis revealed that CAP-H2 protein levels are increased in mitosis, but not in G2 phase in a Plk1 kinase activity-dependent manner (Fig. 2B). These results suggest that CAP-H2 expression is regulated by Plk1 at the early phase of mitosis.

### Plk1 and Cdc20 regulate mitotic CAP-H2 expression

**Figure 3:**
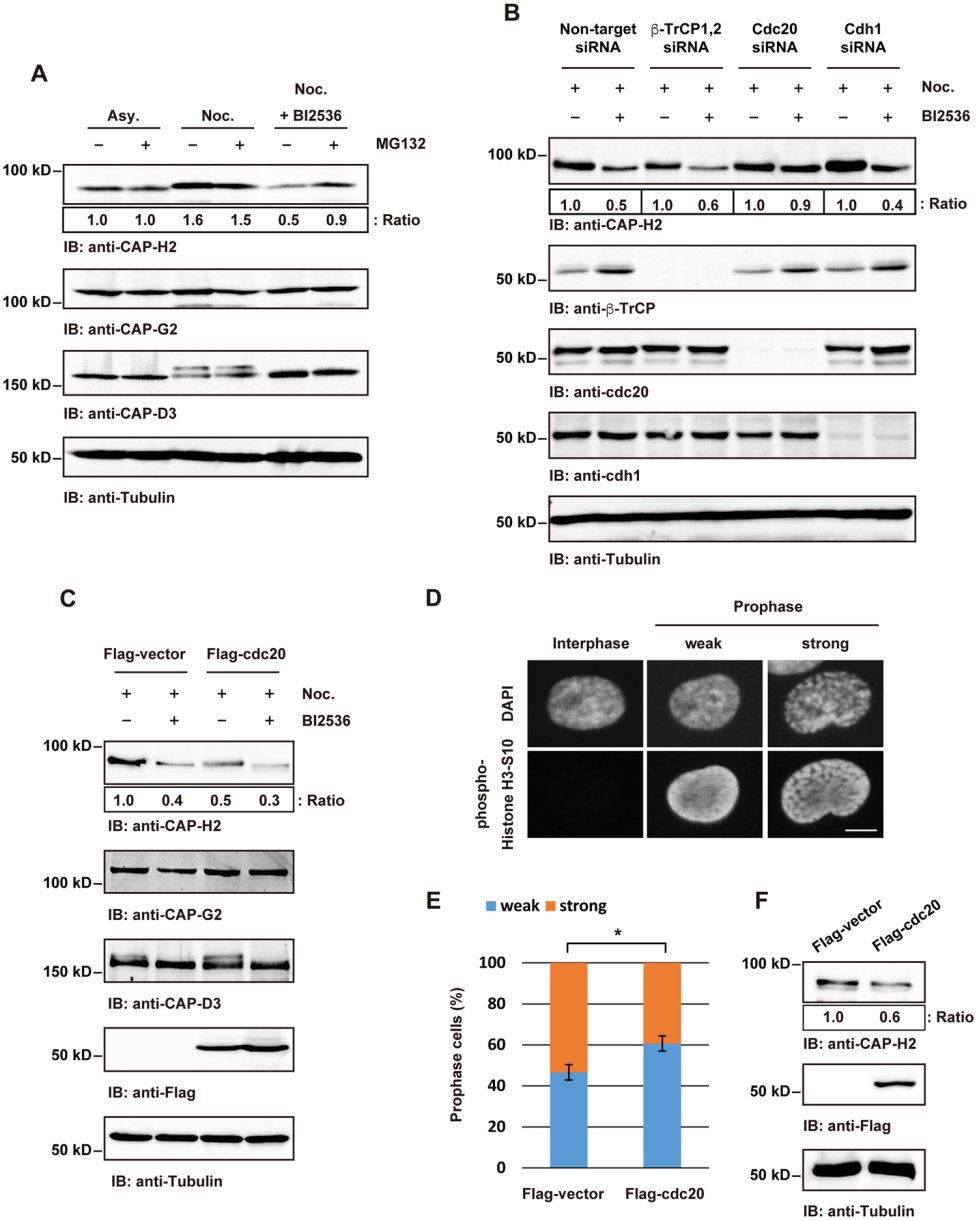
Cdc20 regulates condensin II function by degradation of CAP-H2 during mitosis. (**A**) HeLa cells were synchronized at mitosis by nocodazole with or without BI2536, and then the cells were treated with MG132. Cell lysates were analyzed by immunoblotting with indicating antibodies. A ratio of CAP-H2/Tubulin was determined by the densitometric analysis using Fusion-CAPT-Software. The ratio of CAP-H2/Tubulin in untreated cells was defined as 1.0. (**B**) HeLa cells were transfected with non-target siRNA, β-TrCP1/2 siRNA, Cdc20 siRNA or Cdh1 siRNA. At 24 h after transfection, cells were treated with nocodazole and BI2536 for 16 h. Mitotic cell lysates were immunoblotted with the indicated antibodies. A ratio of CAP-H2/Tubulin was determined by the densitometric analysis using Fusion-CAPT-Software. The ratio of CAP-H2/Tubulin in each of siRNA transfected and nocodazole treated cells was defined as 1.0. (**C**) HeLa cells were transfected with Flag-vector or Flag-Cdc20. At 24 h after transfection, cells were treated with nocodazole and BI2536 for 16 h. Mitotic cell lysates were immunoblotted with the indicated antibodies. A ratio of CAP-H2/Tubulin was determined by the densitometric analysis using Fusion-CAPT-Software. The ratio of CAP-H2/Tubulin in Flag-vector transfected and nocodazole treated cells was defined as 1.0. (**D**) Fixed HeLa cells were stained with anti-phospho-histone H3-Ser10. DNA was stained by DAPI. Degrees of prophase chromosome condensation were defined in the two categories and the representative cells are shown. Scale bar, 10 μm. (**E**) HeLa cells were transfected with Flag-vector or Flag-Cdc20 and synchronized at G1/S phase by thymidine treatment. Cells were then released into the cell cycle. At 9 h after release, cells were fixed and were stained with anti-phospho-histone H3-Ser10 and anti-Flag. The percentage of each category, defined in D, was calculated. n > 100 cells per indicated condition were analyzed. Data represent mean ± SD from three independent experiments. Statistical analysis was performed with the Student’s t test (*P < 0.05). (**F**) The cells were transfected and synchronized as described in E. Cell lysates were analyzed by immunoblot analysis using indicated antibodies. A ratio of CAP-H2/Tubulin was determined by the densitometric analysis using Fusion-CAPT-Software. The ratio of CAP-H2/Tubulin in Flag-vector transfected cells was defined as 1.0.

To investigate whether CAP-H2 is degraded by an ubiquitin-proteasome machinery, HeLa cells were treated with nocodazole, BI2536 and the 26S proteasome inhibitor MG132. Immunoblot analysis revealed that the reduction in mitotic CAP-H2 following Plk1 inhibition is rescued by MG132 (Fig. 3A), indicating that the expression levels of CAP-H2 are controlled by Plk1 phosphorylation and proteasome system during mitosis. Previous studies have demonstrated that the E3 ubiquitin ligase complexes, SCF/β-TrCP, APC/Cdc20, and APC/Cdh1 play pivotal roles in the progression from G2 phase to mitosis^11, 15^. In this context, we next examined whether these E3 ligase complexes participate in degradation of CAP-H2. HeLa cells were transfected with siRNA targeting for β-TrCP, Cdc20, or Cdh1 followed by nocodazole with or without BI2536 treatment. Immunoblot analysis revealed that Plk1 inhibition leads to a reduction of CAP-H2 in control siRNA, β-TrCP siRNA, or Cdh1 siRNA-transfected cells (Fig. 3B). In contrast, the abundance of CAP-H2 remained unchanged in cells silenced for Cdc20 (Fig. 3B). Moreover, the overexpression of Cdc20 led to attenuation of CAP-H2 but not CAP-G2 or CAP-D3 in mitotic cells (Fig. 3C). Collectively, these results suggest that CAP-H2 is downregulated by Cdc20-mediated degradation. To confirm these findings, we next performed co-immunoprecipitation assay using lysates obtained from GFP-CAP-H2 transfected HeLa cells. Immunoprecipitation and immunoblot analyses demonstrated that GFP-CAP-H2 interacts with endogenous Cdc20 (Supplemental Fig. S2A). To investigate if APC/Cdc20 ubiquitinates CAP-H2, we performed *in vitro* ubiquitination assays. APC E3 ligase complex was obtained from nocodazole-treated HeLa cells that were transfected with non-target siRNA or Cdc20 siRNA (Supplemental Fig. S2B) and the APC complex was incubated with recombinant CAP-H2. Immunoblot analysis demonstrated that ubiquitnated CAP-H2 is increased by APC/Cdc20 (Supplemental Fig. S2C). Depletion of Cdc20 from APC complex slightly repressed ubiquitination of CAP-H2 (Supplemental Fig. S2C), suggesting that CAP-H2 is a potential substrate for APC/Cdc20 complex.

A previous study has demonstrated that prophase chromosome condensation is reduced by the depletion of a condensin II subunit^16^. In this context, we investigated whether Cdc20 affects chromosomal condensation during prophase. HeLa cells transfected with Flag-vector or Flag-Cdc20 vector were synchronized at G1/S phase and then were released into cell cycle. As reported previously^8, 16^, prophase was identified by the phosphorylation of histone H3 at Ser10 and by an intact nuclear envelope. The prophase chromosome condensation was defined as two categories. In the “weak” category, DAPI and the phospho-histone H3 signal were uniformly distributed in the nucleus (Fig. 3D, weak). In the “strong” category, a thread-like structure was clearly visible by both DAPI and phospho-histone H3 staining (Fig. 3D, strong). As compared to control cells, the degree of prophase chromosome condensation was lesser in Flag-Cdc20 transfected cells (Fig. 3E and F). These findings suggest that overexpression of Cdc20 causes a defect in prophase chromosome condensation by CAP-H2 degradation.

### Plk1 phosphorylates CAP-H2 at Ser288

**Figure 4:**
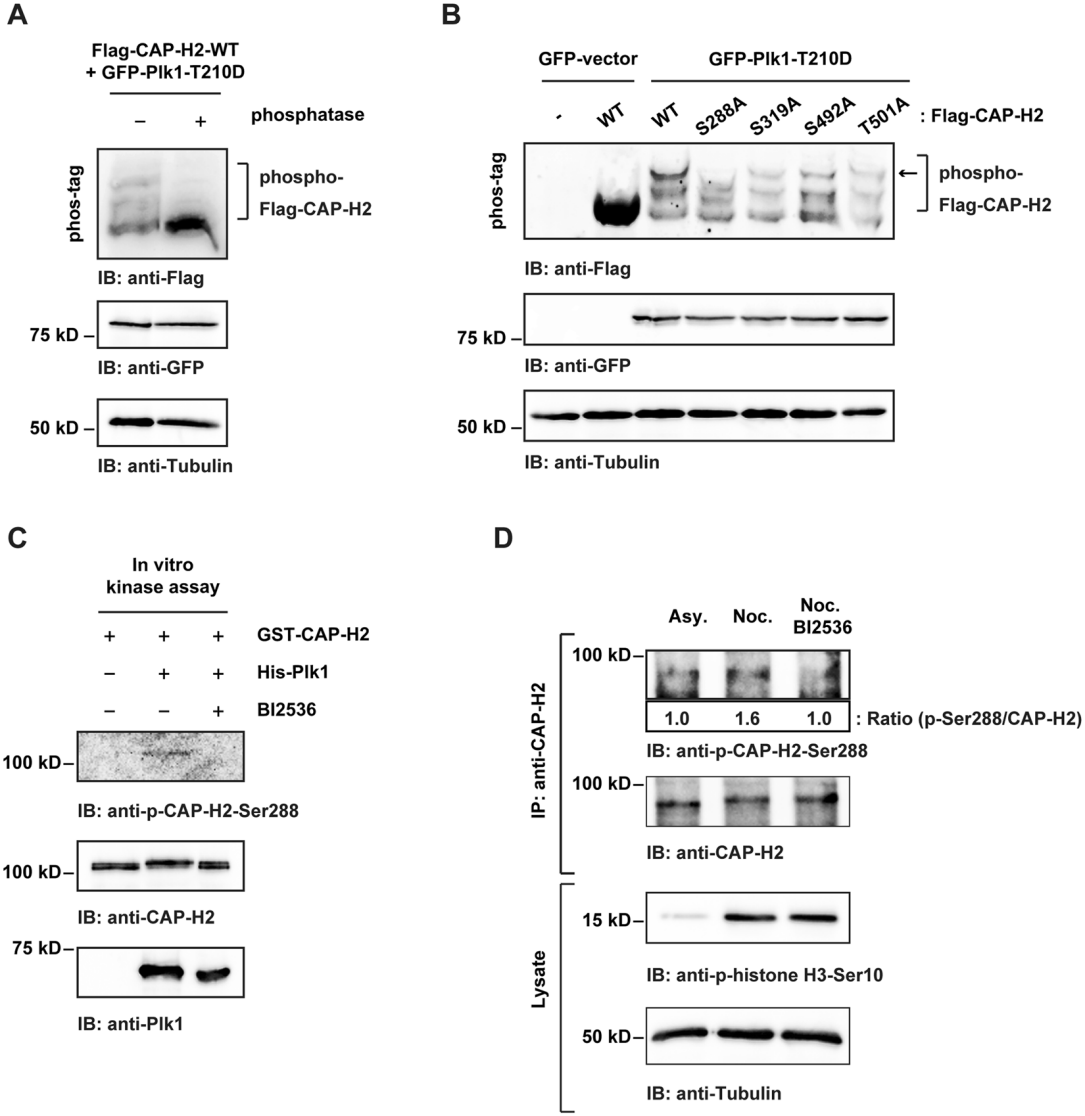
Identification of Ser288 of CAP-H2 as a Plk1 phosphorylation site. (**A**) 293 cells were co-transfected with Flag-CAP-H2 and GFP-Plk1-T210D. Cell lysates were incubated in the absence or presence of lambda protein phosphatase. The lysates were subjected to phos-tag SDS-PAGE and immunoblot analysis. (**B**) 293 cells were co-transfected with indicated plasmids. Cell lysates were subjected to phos-tag SDS-PAGE and immunoblot analysis using indicated antibodies. Hyper phosphorylated form of Flag-CAP-H2 (red arrow) was diminished in S288A mutant. (**C**) Recombinant GST-CAP-H2 was incubated with ATP in the absence or presence of His-Plk1 and BI2536. The reaction products were analyzed by immunoblotting with the indicated antibodies. (**D**) HeLa cells were arrested at mitosis by nocodazole and then were treated with BI2536 for 2 h. The lysates were subjected to immunoprecipitation with anti-CAP-H2. The lysates and immuneprecipitates were immunoblotted with the indicated antibodies. A ratio of p-CAP-H2-Ser288/CAP-H2 was determined by the densitometric analysis using Fusion-CAPT-Software. The ratio of p-CAP-H2-Ser288/CAP-H2 in asynchronous cells was defined as 1.0.

As shown previously, the abundance of CAP-H2 is correlated with Plk1 kinase activity (Figs. 1 and 2). In this regard, we speculated that Plk1 contributes to stabilization of CAP-H2 by phosphorylation. To identify the phosphorylation sites of CAP-H2 by Plk1, we performed *in vitro* kinase assays using recombinant proteins (Supplemental Fig. S3A), and the samples were then applied for the mass spectrometry analysis. The mass spectral data revealed that Ser288, Ser319, Ser492, and Thr501 of CAP-H2 are potential phosphorylation residues by Plk1 *in vitro* (Table 1). To analyze further, 293 cells were co-transfected with the wild type (WT) or the non-phosphorylatable mutant of Flag-CAP-H2, in which Ser288, Ser319, Ser492 and Thr501 are substituted to alanine, and a constitutive active form of GFP-Plk1 (GFP-Plk1-T210D). The phosphorylated Flag-CAP-H2 was separated using phos-tag SDS-PAGE by which phosphorylation status can be detected as mobility shifts (Fig. 4A). Immunoblot analysis demonstrated that Flag-CAP-H2-WT is phosphorylated by GFP-Plk1-T210D (Fig. 4A and B). Although the series of Flag-CAP-H2 mutants were also phosphorylated by Plk1, the hyper-phosphorylated form of Flag-CAP-H2 was abolished only in the Ser288A mutant (Fig. 4B). These findings indicated that Plk1 phosphorylates CAP-H2 at Ser288 in cells. To confirm that Plk1 phosphorylates Ser288, we raised a specific antibody against phosphorylated Ser288. A dot blot analysis using phosphopeptides or non-phosphopeptides of CAP-H2 indicated the specificity of the antibody against Ser288 phosphorylation (Supplemental Fig. S3B). *In vitro* kinase assays demonstrated that Plk1 can directly phosphorylate Ser288 *in vitro* (Fig. 4C). To examine whether Plk1 phosphorylates the endogenous CAP-H2 at Ser288 in mitotic cells, HeLa cells were arrested at mitosis by nocodazole and then were treated with BI2536. Immunoblot analysis demonstrated that the phosphorylation of Ser288 is increased in mitosis (Fig. 4D and Supplemental Fig. S3C) whereas Plk1 inhibition diminished the Ser288 phosphorylation during mitosis (Fig. 4D). These results demonstrate that Plk1 phosphorylates CAP-H2 at Ser288 in mitosis.

**Table 1:** Mass spectrometry analysis of CAP-H2 phosphorylation by Plk1 *in vitro*.

Phospho- site	Peptide sequence	Plk1 phosphorylation
S95	QLS(p)SVQEDR	−
S284	ESRS(p)PQQSAALPR	−
S288	SPQQS(p)AALPR	+
S319	ETPDPWQS(p)LDPFDSLESKPFK	+
S376	LQDFHQWYLAAYADHADS(p)R	−
S385	KGPS(p)FADMEVLYWTHVK	−
S492	FVQETELS(p)QR	+
T501	IRDWEDT(p)VQPLLQEQEQHVPFDIHTYGDQLVSR	+

All of identified phospho-peptides are shown. +means the phosphorylated site that is increased by Plk1.

### Ser288 phosphorylation is required for the fluctuation of CAP-H2

**Figure 5:**
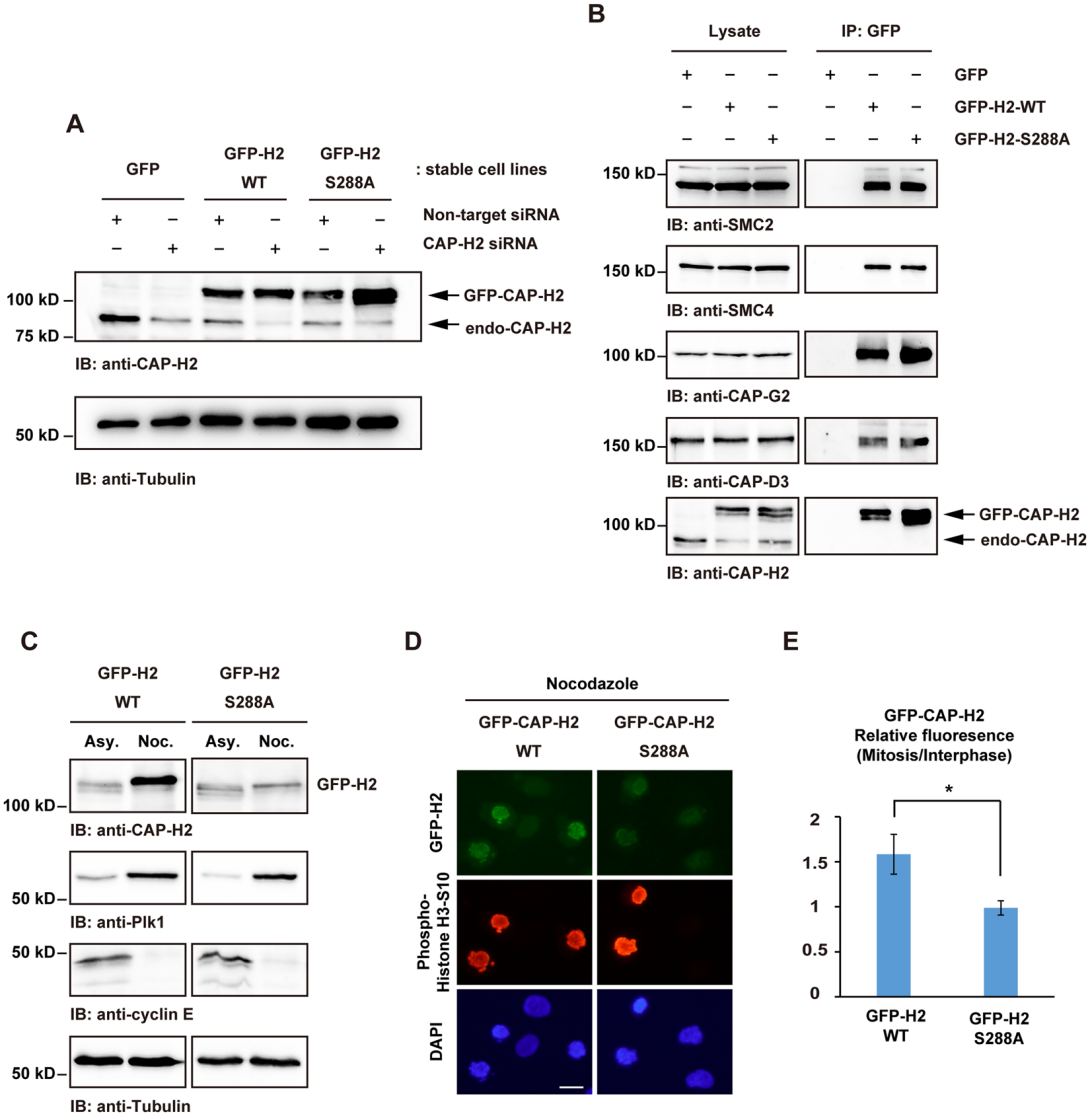
S288 phosphorylation regulates the fluctuation of CAP-H2 in mitosis. (**A**) HeLa cells stably expressing GFP (GFP), GFP-CAP-H2-WT (WT), or GFP-CAP-H2-S288A (S288A) were transfected with non-target siRNA or CAP-H2 siRNA. Lysates were immunoblotted with indicated antibodies. (**B**) Lysates from GFP, WT, or S288A cell lines were immunoprecipitated with anti-GFP and were immunoblotted with the indicated antibodies. (**C**) WT or S288A stable cell lines were transfected with non-target siRNA or CAP-H2 siRNA followed by nocodazole treatment. Cell lysates were immunoblotted with the indicated antibodies. (**D**) WT or S288A stable cell lines were treated with nocodazole and then fixed. Fixed cells were stained with anti-phospho-histone-H3 Ser10. DNA was stained by DAPI. Scale bar, 20 μm. (**E**) The mean fluorescence of mitotic GFP-CAP-H2 was normalized by fluorescence of interphase GFP-CAP-H2 in WT or S288A stable cell lines. n > 30 mitotic cells per indicated condition were analyzed. Data represent mean ± SD from three independent experiments. Statistical analysis was performed with the Student’s t test (*P < 0.05).

To further analyze the significance for the Plk1-mediated phosphorylation of CAP-H2, we generated a cell line that stably express GFP-CAP-H2-S288A mutant. Of note, GFP-CAP-H2 was a resistant form against CAP-H2 siRNA (Fig. 5A). Both the wild-type and S288A mutant CAP-H2 could form condensin complexes in cells (Fig. 5B). Furthermore, CAP-H2 at Ser288 was phosphorylated in wild-type, but not in the S288A mutant during mitosis (Supplemental Fig. S4A). To determine if Ser288 phosphorylation is required for the fluctuation of CAP-H2 expression in mitosis, GFP-CAP-H2 stable cell lines were synchronized at mitosis. Immunoblot analysis revealed that the mitotic expression levels of GFP-CAP-H2 are upregulated in wild-type but not in the S288A mutant (Fig. 5C). Fluorescence microscopic analysis showed that the GFP-CAP-H2-WT increases in mitotic cells; however, there is little if any changes in the abundance of S288A mutant between in interphase and in mitosis (Fig. 5D and E). Moreover, the ubiquitination of GFP-CAP-H2 was increased by S288A mutation in mitotic cells (Supplemental Fig. S4B). These results suggest that the phosphorylation of Ser288 is associated with regulating CAP-H2 expression levels during mitosis.

### Condensin II function is regulated by Plk1 phosphorylation of CAP-H2 at Ser288 in the early phase of mitosis

**Figure 6:**
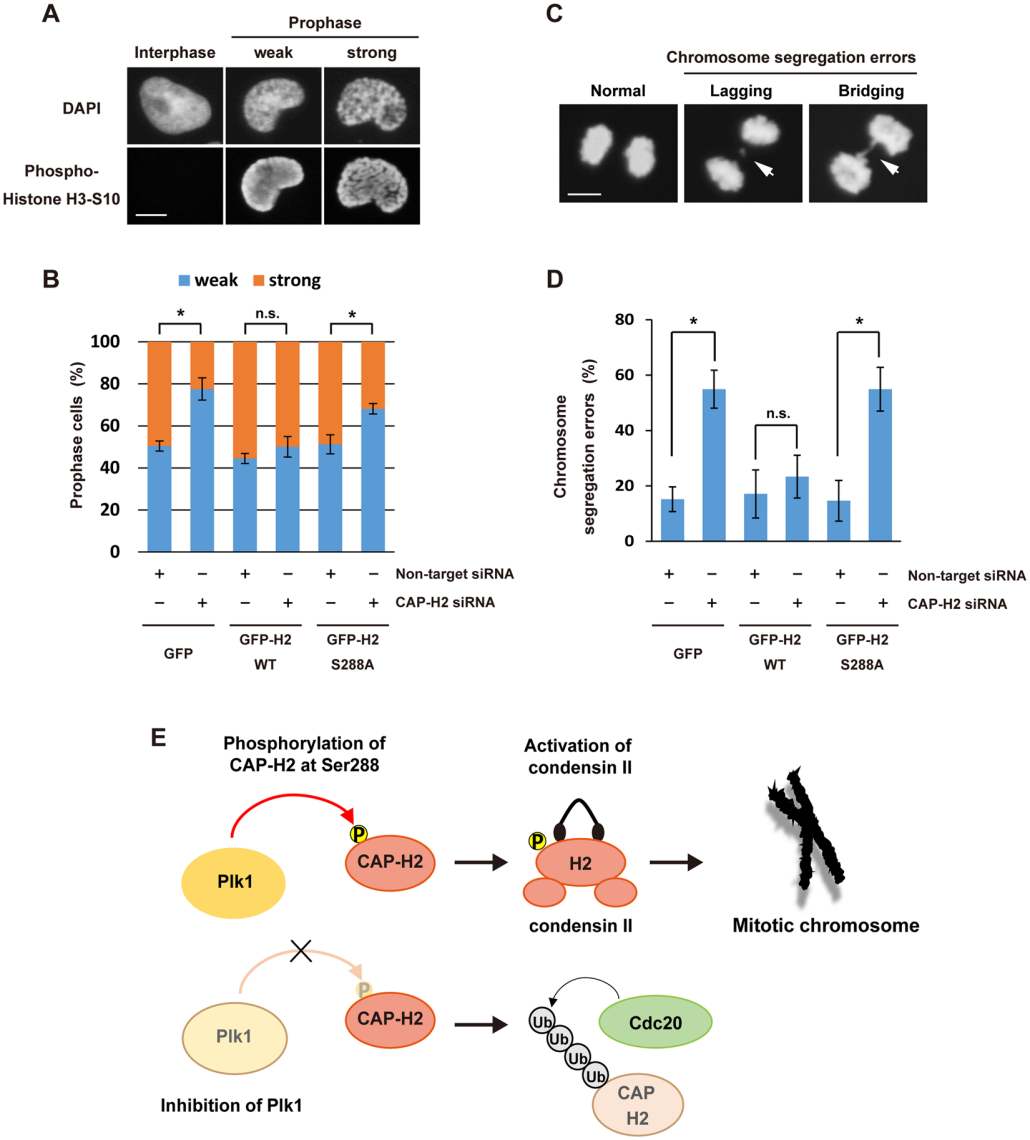
S288 phosphorylation is required for the condensin II functions during mitosis. (**A**) GFP, GFP-CAP-H2-WT or GFP-CAP-H2-S288A stable cell lines were transfected with non-target siRNA or CAP-H2 siRNA. The cells were fixed and stained with anti-phospho-histone H3-Ser10. DNA was stained by DAPI. Degrees of prophase chromosome condensation were defined in the two categories and the representative cells are shown. Scale bar, 10 μm. (**B**) GFP, GFP-CAP-H2-WT, or GFP-CAP-H2-S288A stable cell lines were transfected with non-target siRNA or CAP-H2 siRNA and then fixed. The percentage of each category, defined in A, was calculated. n > 100 cells per indicated condition were analyzed. Data represent mean ± SD from three independent experiments. Statistical analysis was performed with the Student’s t test (*P < 0.05). (**C**) GFP, GFP-CAP-H2-WT, or GFP-CAP-H2-S288A stable cell lines were transfected with non-target siRNA or CAP-H2 siRNA. The cells were fixed and stained with anti-phospho-histone H3-Ser10. DNA was stained by DAPI. The anaphase cells were assessed for the presence of chromosome segregation errors, lagging and/or bridging chromosomes. The representative cells are shown. Scale bar, 10 μm. (**D**) The percentage of chromosome segregation errors, as described in C, was calculated. n > 100 cells per indicated condition were analyzed. Data represent mean ± SD from three independent experiments. Statistical analysis was performed with the Student’s t test (*P < 0.05). (**E**) A model of condensin II regulation by Plk1 kinase. Plk1 phosphorylates CAP-H2 at Ser288 in mitosis. Ser288 phosphorylation is concerned with the fluctuation of CAP-H2 and regulates condensin II functions during mitosis.

To examine whether Plk1-mediated Ser288 phosphorylation regulates condensin II functions, we analyzed chromosome condensation during prophase in GFP-CAP-H2 stable cell lines (Fig. 6A). As reported previously^8^, a reduction in prophase chromosome condensation was observed in CAP-H2-depleted control cells (Fig. 6B). Importantly, the expression of GFP-CAP-H2-WT rescued this reduction whereas there was a little effect by the S288A mutant (Fig. 6B). Given the finding that dysregulation of condensin II causes a defect in mitotic chromosome segregation^16, 17^, we further analyzed the chromosome segregation failure in GFP-CAP-H2 stable cell lines. The results demonstrated that chromosome segregation errors, such as lagging chromosome and chromosome bridging, were induced by depletion of CAP-H2 in control cells (Fig. 6C and D). Furthermore, the defects were rescued by GFP-CAP-H2-WT, but not by the S288A mutant (Fig. 6C and D). These results indicate that Ser288 phosphorylation of CAP-H2 is required for mitotic chromosome segregation. Taken together, these findings support a model in which Plk1 phosphorylates CAP-H2 at Ser288 to regulate fluctuation of CAP-H2 and condensin II function during mitosis (Fig. 6E).

## Discussion

Our results show that the abundance of CAP-H2 is fluctuated during the cell cycle and Plk1 phosphorylation of CAP-H2 at Ser288 is a key modification to regulate proper condensin II function in mitosis. We also demonstrate that inhibition of Plk1 kinase activity promotes Cdc20-mediated degradation of CAP-H2 (Fig. 3B). Importantly, a previous study has reported that overexpression of Cdc20 causes a defect in mitotic chromosome structure^18^. In this context, we observed a decreased expression of CAP-H2 and a reduction of prophase chromosomal condensation in Flag-Cdc20 transfected cells (Fig. 3C and E). A previous study has reported that in interphase Drosophila cells, CAP-H2 is suppressed by SCF/Slimb-mediated ubiquitination; however, the binding domain of Slimb in CAP-H2 is not conserved in other organisms^10^. Our results show that CAP-H2 is not affected by depletion of β-TrCP, an ortholog of Drosophila Slimb, in mitotic HeLa cells (Fig. 3B). In this context, Cdc20 but not β-TrCP regulates CAP-H2 expression levels in mitotic HeLa cells. However, it is important to point out that APC/Cdc20 activity is restricted during the early phase of mitosis. A recent study has shown that the E3 ubiquitin ligase Parkin interacts with Cdc20 and Parkin/Cdc20 mediates the degradation of several mitotic substrates in parallel with APC/Cdc20^19^. In this context, Cdc20 may act as an activator for not only APC but also other E3 ligases. Although our results suggested that CAP-H2 level is regulated by Cdc20, a detailed analysis is necessary to reveal a mechanism for Cdc20-mediated CAP-H2 degradation.

Non-phosphorylatable mutant of Ser288 also prevents increasing CAP-H2 protein levels and the S288A mutant is subjected to ubiquitination in mitotic cells (Fig. 5C and Supplemental Fig. S4B), suggesting that Ser288 phosphorylation contributes to stabilization of CAP-H2. In this regard, the phospho-mimicking S288D mutation would lead increased expression of CAP-H2 throughout the cell cycle. A previous study has shown that overexpression of CAP-H2 causes dense chromatin compaction in interphase Drosophila cells^10^. In addition, a recent study has reported that condensin II promotes gene expression during interphase^20^. These findings thus imply the possibility that dysregulated CAP-H2 protein levels could affect chromosomal organization and gene expressions. Our results showed that the Ser288 of CAP-H2 is phosphorylated not only in mitotic cells but also in asynchronous cells (Fig. 4D). This observation implies the possibility that Ser288 phosphorylation could contribute to the stabilization of CAP-H2 in interphase. Since the activity of Plk1 is increased during the cell cycle from G2 phase to mitosis^11, 21^, the other kinase would be involved in regulating interphase CAP-H2. A recent study has revealed that Ycg1, the Cap-G subunit of budding yeast condensin, is degraded by ubiquitin-proteasome system and a condensin function is restricted during interphase^22^. Furthermore, human condensin II subunits are reported to undergo proteolytic degradation^23^. In this regard, condensin function would be regulated at protein levels by a cell cycle-dependent manner.

Previous studies have demonstrated the molecular mechanisms by which phosphorylation regulates the localization and the activation of condensin II during mitosis. The current study shows a novel mechanism in which the abundance of CAP-H2 is controlled by Plk1 phosphorylation. Dysregulation of chromosomal organization causes several diseases including cancer. A large proportion of cancer cells shows chromosomal aberration and genomic instability. Furthermore, increased nuclear size is common features of cancer cells. Notably, a previous study has reported that condensin II alters nuclear morphology by regulating chromatin organization, and thus dysregulation of condensin II causes abnormal nuclear size^24^. Taken together with the findings that Plk1 and Cdc20 are overexpressed in several tumors^25, 26^, the abundance of CAP-H2 and condensin II function would be dysregulated in cancer cells. Although condensin II function in cancer cells remains largely unclear, condensin II could play critical roles for controlling aberrant chromosomes in cancer cells. To address these possibilities, further analysis is definitely required.

## Methods

### Cell culture and cell synchronization

HeLa cells, 293 cells and RPE1 cells were cultured in DMEM containing 10% FBS, penicillin and streptomycin. Cells were maintained at 37 °C in 5% CO_2_. To synchronize at mitosis, cells were treated with 200 ng/ml nocodazole (Sigma-Aldrich) for 14 h. Mitotic cells were collected in suspension by gentle shaking cells off the culture dishes. To synchronize at the G1/S phase, cells were synchronized using a double thymidine block method. Cells were treated with 2 mM thymidine (Wako) for 14 h, washed twice with fresh medium and released for 10 h. Thymidine was added again to a final concentration of 2 mM to synchronize cells at the G1/S phase. To synchronize at the G2 phase, cells were synchronized by double thymidine methods and then released into medium containing 9 µM RO3306 (Sigma-Aldrich) for 12 h. Plk1 inhibitor BI2536 (Chemitek) and proteasome inhibitor MG132 (Calbiochem) were used with 100 nM and 5 µM respectively.

### Preparation of a phospho-specific antibody

To generate a phospho-specific antibody against Ser288-phosphorylated CAP-H2, phosphopeptides (RSPQQ(pS)AALPR) were used to immunize rabbits (Eurofins). The anti-serum was affinity-purified against the phosphorylated peptides.

### Immunoblot and immunoprecipitation

Cells were harvested, washed in PBS, and resuspended in SDS-lysis buffer (50 mM Tris-HCl (pH 7.6), 150 mM NaCl, 1 mM PMSF, 1 mM DTT, 10 µg/ml aprotinin, 1 µg/ml leupeptin, 1 µg/ml pepstatin A, 1% SDS, and 1% NP-40) with phosphatase inhibitor (10 mM NaF and 1 mM Na_3_VO_4_) and sonicated. After centrifugation, the supernatants were isolated and used as cell lysates. For the immunoprecipitation, cells were harvested and resuspended in lysis buffer (50 mM Tris-HCl (pH 7.6), 150 mM NaCl, 1 mM PMSF, 1 mM DTT, 10 µg/ml aprotinin, 1 µg/ml leupeptin, 1 µg/ml pepstatin A, and 0.1% NP-40) with phosphatase inhibitor (10 mM NaF and 1 mM Na_3_VO_4_). Lysates were incubated with mouse anti-APC3 (BD Bioscience), or rabbit anti-CAP-H2 (Bethyl Laboratories) for 1 h at 4 °C. Then, the solutions were incubated with Protein G-Sepharose or Protein A-Sepharose CL-4B (GE Healthcare Bio-Sciences) for 2 h at 4 °C. For the immunoprecipitation of Flag-tagged proteins, lysates were incubated with anti-Flag agarose (Sigma-Aldrich). For the immunoprecipitation of GFP-tagged proteins, lysates were incubated with anti-GFP agarose (Medical & Biological Laboratories). The beads were washed three times in lysis buffer. The lysates and immunoprecipitated proteins were boiled for 5 min, separated by SDS-PAGE or phos-tag (Wako) SDS-PAGE, and transferred onto nitrocellulose membranes. The membranes were blocked with Blocking One (Nacalai Tesque), washed three times in TBS with 0.05% Tween-20, incubated with rabbit anti-SMC2 (Bethyl Laboratories), rabbit anti-SMC4 (Bethyl Laboratories), rabbit anti-CAP-H2 (Bethyl Laboratories), rabbit anti-CAP-G2 (Bethyl Laboratories), rabbit anti-CAP-D3 (Bethyl Laboratories), rabbit anti-phospho-histone H3-Ser10 (Millipore), mouse anti-Plk1 (Santa Cruz Biotechnology), mouse anti-APC3 (BD Bioscience), rabbit anti-TrCP (Cell Signaling Technology), rabbit anti-Cdc20 (Santa Cruz Biotechnology), mouse anti-cdh1 (Calbiochem), mouse anti-Cyclin E (Santa Cruz Biotechnology), mouse anti-Tubulin (Sigma-Aldrich), mouse anti-GFP (Nacalai Tesque), rabbit anti-Flag (Cell Signaling Technology), mouse anti-GST (Nacalai Tesque), or rabbit anti-phospho-CAP-H2-Ser288. Membranes were then washed three times in TBS with 0.05% Tween-20, and incubated with peroxidase-conjugated anti-rabbit IgG (Santa Cruz Biotechnology) or peroxidase-conjugated anti-mouse IgG (Santa Cruz Biotechnology). Immune complexes were visualized by using a Western Lightning Plus-ECL (PerkinElmer).

### Real-time RT-PCR analysis

Isolation of total RNA from cells was performed using TRIsure (Nippon Genetics) according to the manufacturer’s instructions. For real-time RT-PCR analysis, total RNA was reverse transcribed into cDNA using a PrimeScript 1st strand cDNA Synthesis Kit (Takara) according to the manufacturer’s protocol. The PCR reaction was performed by using KAPA SYBR FAST ABI Prism 2X qPCR Master Mix (Nippon Genetics) according to the instruction manual. Primer sequences are described as follows: CAP-H2 5′-CTGGACCCCTTTGACTCCTT-3′ and 5′-CTTCCTCTTGCGCTTCTGTC-3′; and GAPDH, 5′-TCAAGGCTGAGAACGGGAAG-3′ and 5′-ATGGTGGTGAAGACGCCAGT-3′.

### Fluorescence microscopic analysis

Cells cultured in chamber slides were fixed with 4% paraformaldehyde. Fixed cells were permeabilized with 1% Triton X-100 in PBS and incubated with 10% goat-serum in PBS for 1 h. Cells were incubated with mouse anti-Flag (Trans Genic Inc) or rabbit anti-phospho-histone H3-Ser10 (Millipore) followed by a reaction with fluorescein isothiocyanate- or tetramethyl rhodamine isothiocyanate-conjugated secondary antibodies. DNA was stained with 4,6-diamidino-2-phenylindole (DAPI). Fixed cells were imaged at room temperature using an All-in-one type fluorescence microscope (Bio-Zero BZ-8000; Keyence) equipped with a Plan App 20 × /0.75 NA objective lens (Nikon). Images were acquired with BZ Analyzer Software (Keyence). GFP-CAP-H2 signal was quantified using ImageJ software (National Institutes of Health). Imaging data were processed using Photoshop (Adobe).

### *In vitro* kinase assay

Recombinant GST-CAP-H2 (Abnova) was incubated in kinase buffer (20 mM HEPES, 10 mM MgCl_2_, 0.1 mM Na_3_VO_4_, and 2 mM DTT) with His-Plk1 (Sigma-Aldrich) and ATP for 20 min. The reaction products were subjected to mass spectrometric analysis or immunoblot analysis.

### siRNA transfections

The transfection of siRNA was performed using Lipofectamine RNAiMAX (Invitrogen) according to the manufacturer’s instructions. The sequences of siRNAs were as follows: Plk1 siRNA, 5′-gggcggcuuugccaagugctt-3′ and 5′-gcacuuggcaaagccgccctt-3′; β-TrCP1,2 siRNA, 5′-aaguggaauuuguggaacauctt-3′ and 5′-gauguuccacaaauuccacuutt-3′; Cdc20 siRNA, 5′-cggaagaccugccguuacauutt-3′ and 5′-aauguaacggcaggucuuccgtt-3′; Cdh1 siRNA, 5′-ugagaagucucccagucagtt-3′ and 5′-cugacugggagacuucucatt-3′; and CAP-H2 siRNA, 5′-gcugcaggacuuccaccagtt-3′ and 5′-cugguggaaguccugcagctt-3′.

### Plasmid construction and stable cell lines

CAP-H2, Plk1 and Cdc20 cDNA was cloned into pEGFPC1 vector or pcDNA3-Flag vector. Various mutations were introduced by site-directed mutagenesis. siRNA-resistant forms of CAP-H2 were generated by introducing silent mutations in the targeting regions for CAP-H2 siRNA. To generate stable cell lines that express GFP-CAP-H2, HeLa cells were transfected with a plasmid encoding GFP-CAP-H2 using the X-tremeGENE 9 DNA transfection reagent (Roche). Stably expressing cell clones were selected by culture with medium containing G418.

### Mass spectrometric analysis

To identify phosphorylation sites on CAP-H2, a two-dimensional image-converted analysis of liquid chromatography and mass spectrometry (2DICAL) shotgun proteomics analysis was performed as described previously^8, 27^.

### *In vitro* ubiquitination assay

Recombinant GST-CAP-H2 was incubated in ubiquitination buffer (50 mM Tris-HCl, 5 mM MgCl_2_, 2 mM DTT, and 4 mM ATP) with ubiquitin (Boston Biochem), ubiquitin Activating Enzyme (E1) (Sigma-Aldrich), UbcH10 (R&D Systems), and APC complex for 60 min. APC complex was obtained from HeLa cell lysates by immunoprecipitation with anti-APC3 antibodies (BD Bioscience). The reaction products were subjected to immunoblot analysis.

No datasets were generated or analyzed during the current study.

## Electronic supplementary material

Supplementary information
