# Supplementary material for: Plk1 phosphorylation of CAP-H2 triggers chromosome condensation by condensin II at the early phase of mitosis
Source: Sci Rep. 2017 Jul 17;7:5583. doi: 10.1038/s41598-017-05986-7 (PMC5514044; doi:10.1038/s41598-017-05986-7)
Supplement: Supplementary file 1 — Supplementary information [file 41598_2017_5986_MOESM1_ESM.pdf]

Plk1 phosphorylation of CAP-H2 triggers chromosome condensation by condensin II at the early phase of mitosis

Yuya Kagami[1], Masaya Ono[2], and Kiyotsugu Yoshida[1]*

[1]Department of Biochemistry, Jikei University Schools of Medicine, 3-25-8 Nishi-shinbashi, Minato-ku, Tokyo, 105-8461, Japan; [2]Chemotherapy Division and Cancer Proteomics Project, National Cancer Center Research Institute, 5-1-1 Tsukiji, Chuo-ku, Tokyo, 104-0045, Japan.

Legends to supplemental figures

Supplemental figure S1. Depletion of Plk1 reduces CAP-H2 protein levels in mitosis.

(A) HeLa cells were left untreated (Asy.) or treated with nocodazole (Noc.). Cell lysates were incubated with or without lambda phosphatase for 20 min and subjected to immunoblot analysis.

(B) HeLa cells were transfected with non-target siRNA or CAP-H2 siRNA. Cell lysates were analyzed by immunoblotting with indicated antibodies. Total RNA was analyzed by real-time RT-PCR with CAP-H2-specific primer. The value is normalized to GAPDH. Data represent mean ± SD from three independent experiments.

(C) HeLa cells were arrested at G1/S phase by thymidine treatment. The cells were released into medium for 9 h and the mitotic cells were collected by mitotic shake off. Cell lysates were analyzed by immunoblotting with indicated antibodies.

(D) RPE1 cells were arrested at mitosis by nocodazole with or without BI2536. Cell lysates were subjected to immunoblot analysis with the indicated antibodies.

(E) HeLa cells were transfected with non-target siRNA or Plk1 siRNA and then synchronized at G1/S phase by thymidine treatment. The cells were released into medium containing nocodazole for 14 h. The mitotic cell lysates were analyzed by immunoblotting with indicated antibodies. Total RNA was analyzed by real-time RT-PCR with CAP-H2-specific primer. The value is normalized to GAPDH. Data represent mean ± SD from three independent experiments.

Supplemental figure S2. CAP-H2 is a potential substrate for APC/Cdc20 complex.

(A) HeLa cells were transfected with GFP-vector or GFP-CAP-H2. Cell lysates were subjected to immunoprecipitation with anti-GFP. Lysates and immunoprecipitates were analyzed by

immunoblotting with indicated antibodies.

(B) HeLa cells were transfected with non-target siRNA or Cdc20 siRNA followed by nocodazole treatment. Cell lysates were immunoblotted with indicated antibodies.

(C) APC/Cdc20 complex was isolated from the cells described in B by immunoprecipitation with anti-APC3 antibodies. The APC/Cdc20 complex was incubated with recombinant GST-CAP-H2 in the presence of E1, E2, ATP and ubiquitin. The reaction mixtures were immunoblotted with indicated antibodies.

Supplemental figure S3. Identification of Plk1-mediated phosphorylation sites on CAP-H2.

(A) Recombinant GST-CAP-H2 was incubated with ATP in the absence or presence of His-Plk1. The reaction products were analyzed by immunoblotting with the indicated antibodies.

(B) Phosphopeptides (RSPQQ(pS)AALPR) or non-phosphopeptides (RSPQQSAALPR) from CAP-H2 were spotted onto a nitrocellulose membrane and were analyzed by immunoblotting with anti-phospho-CAP-H2-S288 antibodies.

(C) HeLa cells were arrested at mitosis by nocodazole. The lysates were subjected to immunoprecipitation with IgG or anti-CAP-H2. Immunoprecipitates were immunoblotted with the indicated antibodies.

Supplemental figure S4. Assessment of GFP-CAP-H2 stable cell lines.

(A) GFP-CAP-H2-WT or GFP-CAP-H2-S288A stable cell lines were transfected with CAP-H2 siRNA followed by nocodazole treatment. Cell lysates were immunoprecipitated with anti-GFP and were analyzed by immunoblotting with indicated antibodies.

(B) GFP-CAP-H2-WT or GFP-CAP-H2-S288A stable cell lines were transfected with CAP-H2 siRNA and then were synchronized at G1/S phase by thymidine treatment. The cells were released into medium containing RO3306 for 12h and subsequently were released with nocodazole and MG132 for 3h. The cell lysates were immunoprecipitated with anti-GFP and were analyzed by immunoblotting with indicated antibodies.

Figure S1

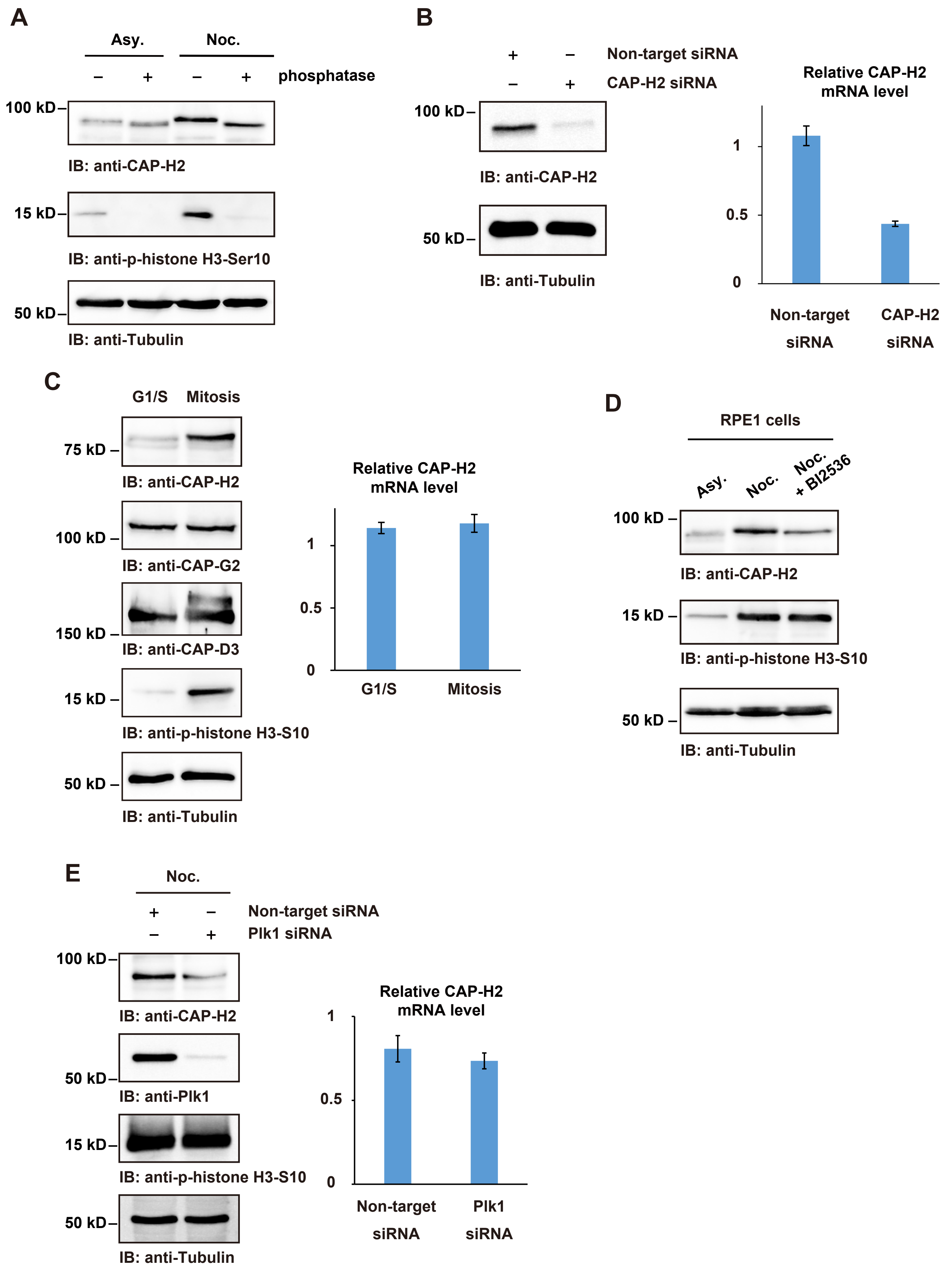
A
Asy.
Noc.
– + – + phosphatase
100 kD
IB: anti-CAP-H2
15 kD
IB: anti-p-histone H3-Ser10
50 kD
IB: anti-Tubulin
B
+ – Non-target siRNA
– + CAP-H2 siRNA
100 kD
IB: anti-CAP-H2
50 kD
IB: anti-Tubulin
Relative CAP-H2
mRNA level
1
0.5
0
Non-target siRNA
CAP-H2 siRNA
C
G1/S
Mitosis
75 kD
IB: anti-CAP-H2
100 kD
IB: anti-CAP-G2
150 kD
IB: anti-CAP-D3
15 kD
IB: anti-p-histone H3-S10
50 kD
IB: anti-Tubulin
Relative CAP-H2
mRNA level
1
0.5
0
G1/S
Mitosis
D
RPE1 cells
Asy.
Noc.
Noc. + BI2536
100 kD
IB: anti-CAP-H2
15 kD
IB: anti-p-histone H3-S10
50 kD
IB: anti-Tubulin
E
Noc.
+ – Non-target siRNA
– + Plk1 siRNA
100 kD
IB: anti-CAP-H2
50 kD
IB: anti-Plk1
15 kD
IB: anti-p-histone H3-S10
50 kD
IB: anti-Tubulin
Relative CAP-H2
mRNA level
1
0.5
0
Non-target siRNA
Plk1 siRNA

Figure S2

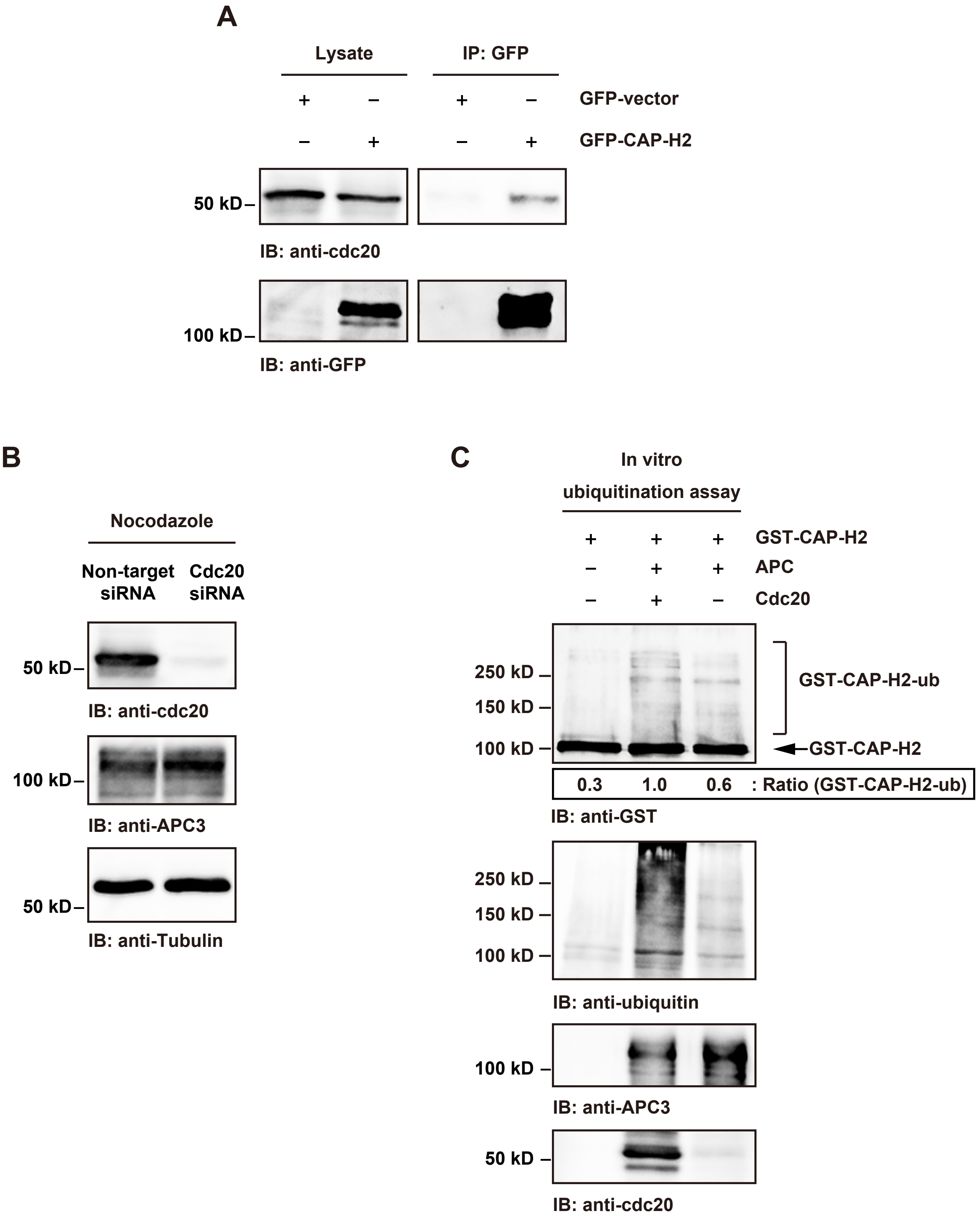
A
Lysate
IP: GFP
+ − + − GFP-vector
− + − + GFP-CAP-H2
50 kD
IB: anti-cdc20
100 kD
IB: anti-GFP
B
Nocodazole
Non-target siRNA
Cdc20 siRNA
50 kD
IB: anti-cdc20
100 kD
IB: anti-APC3
50 kD
IB: anti-Tubulin
C
In vitro ubiquitination assay
+ + + GST-CAP-H2
− + + APC
− + − Cdc20
250 kD
150 kD
100 kD
GST-CAP-H2-ub
GST-CAP-H2
0.3 1.0 0.6 : Ratio (GST-CAP-H2-ub)
IB: anti-GST
IB: anti-ubiquitin
IB: anti-APC3
50 kD
IB: anti-cdc20

Figure S3

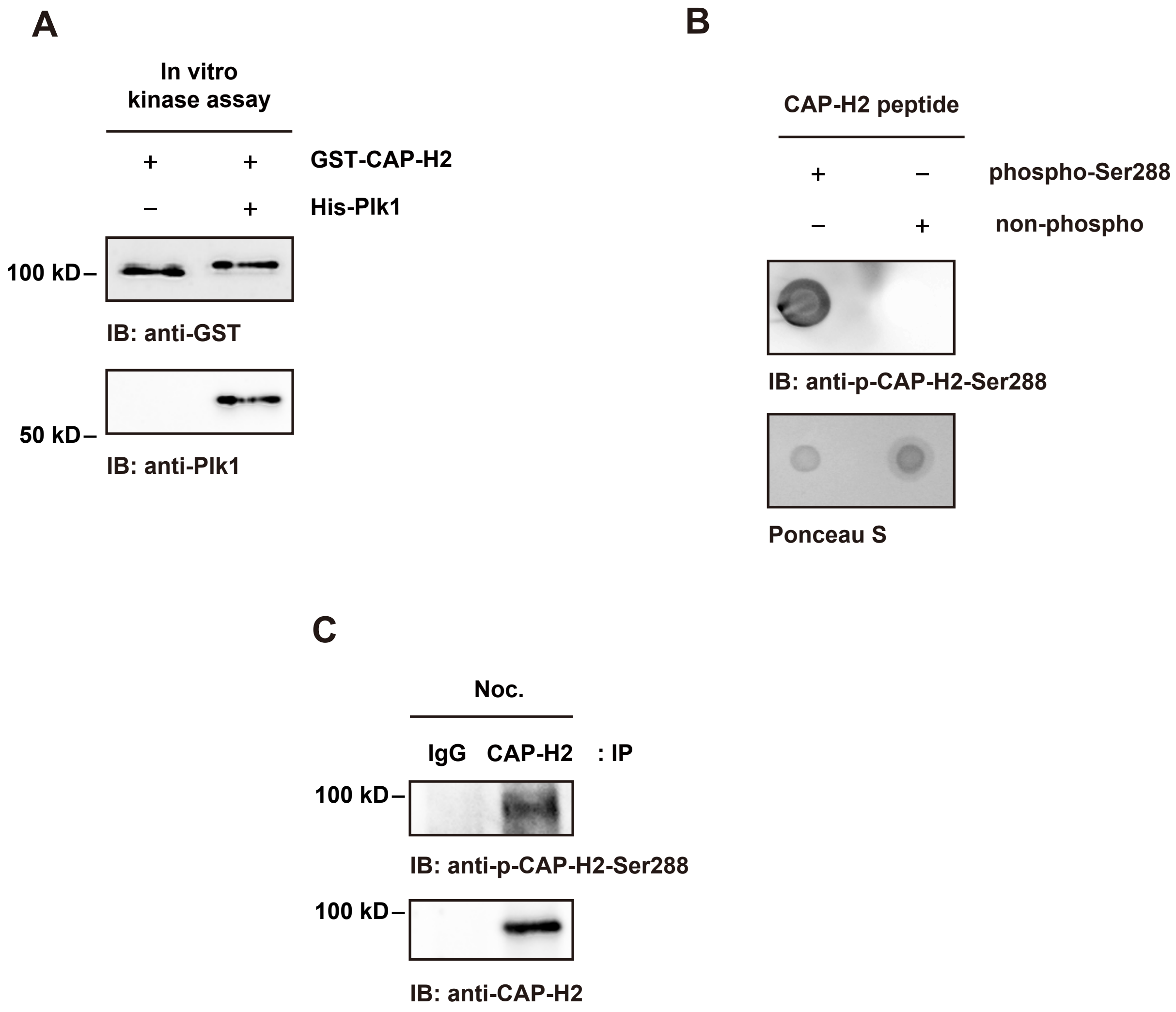
A
In vitro kinase assay
GST-CAP-H2
His-Plk1
100 kD
IB: anti-GST
50 kD
IB: anti-Plk1
B
CAP-H2 peptide
phospho-Ser288
non-phospho
IB: anti-p-CAP-H2-Ser288
Ponceau S
C
Noc.
IgG
CAP-H2
: IP
100 kD
IB: anti-p-CAP-H2-Ser288
100 kD
IB: anti-CAP-H2

Figure S4

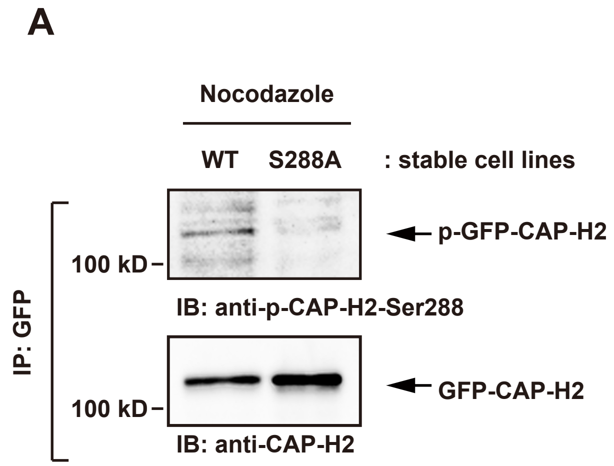
A
Nocodazole
WT
S288A
: stable cell lines
p-GFP-CAP-H2
100 kD
IB: anti-p-CAP-H2-Ser288
IP: GFP
GFP-CAP-H2
100 kD
IB: anti-CAP-H2

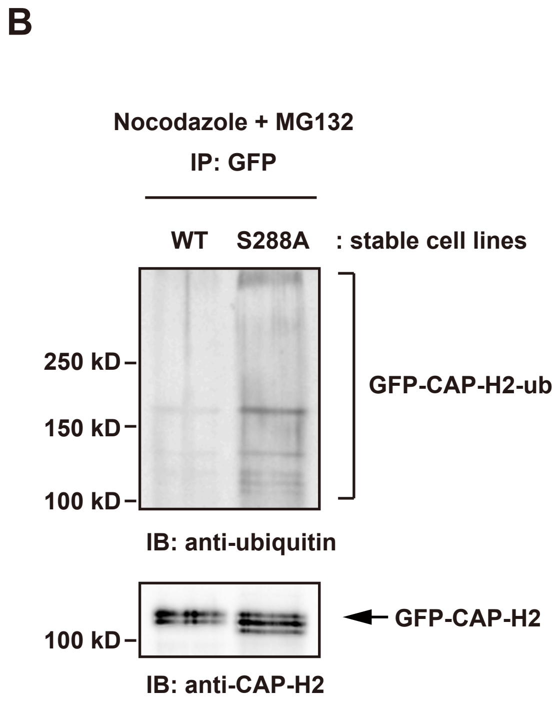
B
Nocodazole + MG132
IP: GFP
WT
S288A
: stable cell lines
250 kD
150 kD
100 kD
GFP-CAP-H2-ub
IB: anti-ubiquitin
GFP-CAP-H2
100 kD
IB: anti-CAP-H2
